# Enhanced Lateral Flow Immunoassay with Double Competition and Two Kinds of Nanoparticles Conjugates for Control of Insecticide Imidacloprid in Honey

**DOI:** 10.3390/bios13050525

**Published:** 2023-05-07

**Authors:** Dmitriy V. Sotnikov, Lyubov V. Barshevskaya, Anatoly V. Zherdev, Boris B. Dzantiev

**Affiliations:** A.N. Bach Institute of Biochemistry, Research Center of Biotechnology of the Russian Academy of Sciences, Leninsky Prospect 33, 119071 Moscow, Russia; sotnikov-d-i@mail.ru (D.V.S.);

**Keywords:** immunochromatography, functionalized nanoparticles, mathematical modeling, neonicotinoids, imidacloprid, food quality

## Abstract

Finding optimal conditions for competitive lateral flow immunoassay is a controversial task. The content of specific antibodies labeled by nanoparticles should be simultaneously high to reach intense signals and low to register an influence on the signals for minimal concentrations of the target analyte. We propose to use two kinds of complexes of gold nanoparticles in the assay, with antigen–protein conjugates and with specific antibodies. The first complex interacts both with immobilized antibodies in the test zone and with antibodies on the surface of the second complex. In this assay, the coloration is enhanced by the binding of two-colored preparations in the test zone, whereas the antigen in the sample inhibits both the binding of the first conjugate with the immobilized antibodies and with the second conjugate. This approach is realized for the detection of insecticide imidacloprid (IMD), an important toxic contaminant connected with the recent global death of bees. The proposed technique expands the working range of the assay, that is, in accordance with its theoretical analysis. The reliable change of coloration intensity is achieved for a 2.3-times-lower concentration of the analyte. The limit of IMD detection is 0.13 ng/mL for tested solutions and 1.2 µg/kg for initial honey samples. The combination of two conjugates doubles the coloration in the absence of the analyte. The developed lateral flow immunoassay is applicable for five-fold-diluted honey samples without extraction, does not require additional stages (all reagents are pre-applied to the test strip), and is implemented in 10 min.

## 1. Introduction

The dominant current trend in the development of analytical techniques is the transition from the use of stationary equipment in specialized laboratories to testing directly at sampling sites [1]. These changes reduce the cost of the assays and the waiting time for the results, and allow for characterizing more samples. Among the analytical methods that meet these requirements and are already widely used for point-of-care testing, lateral flow immunoassay (LFIA) occupies one of the leading places [2]. During LFIA, the contact of the strip containing all the necessary pre-applied reagents and the tested sample initiate all further processes and, in 10–15 min, cause the coloring of certain areas of the strip, which is assessed visually or by a portable detector, and reflects the presence and/or content of the target analyte [3,4,5].

For low-molecular-weight analytes with only one antigenic determinant, competitive formats of LFIA are used, when, typically, the conjugate of the antigen derivative with a protein carrier applied to the test strip and the antigen in the sample compete to bind with antibodies labeled with a colored nanoparticle. However, competitive LFIA is usually less sensitive than other kinds of immunoassays, even using the same reagents [6]. This is connected with the direct immediate registration of the formed immune complexes in the LFIA by the coloration intensities of the bound label. In other methods, for example, in the enzyme-linked immunosorbent assay (ELISA), the catalytic activity of the enzyme label ensures the development of the coloration. As a result of this limitation of the LFIA, when choosing the optimal concentration of the labeled antibodies, we cannot significantly reduce it because this will reduce the detected signal and make the registered LFIA result less reliable. However, we cannot also significantly increase it because then the inhibition of the binding of the labeled antibodies to the antigen–protein conjugate will be recorded only for high concentrations of the antigen.

The main ways to overcome this limitation of the competitive LFIA are the use of labels detected at lower concentrations (e.g., fluorescent or magnetic labels) [7,8] or additional treatment of the binding zone with reagents that provide color development [9,10,11]. However, such changes lead to more complicated and time-consuming testing.

Earlier, we proposed a competitive LFIA with double competition, in which the antibodies against the analyte are immobilized both on the surface of nanoparticles and on the test strip, and a leachable conjugate of a protein with several antigen molecules is applied to the initial part of the test strip. In the absence of the analyte, the [immobilized antibody–analyte–protein conjugate–labeled antibody] complex is formed in the test zone. The antigen in the sample competes to bind with both the immobilized and labeled antibodies. It was demonstrated that the double competition in the LFIA allows the detection of lower concentrations of the antigen [12].

In this work, the double competition is combined with the use of two kinds of complexes of a nanosized label: with specific antibodies and with an analyte–protein conjugate. The additional second preparation interacts with the antigen-binding sites of the antibodies of the first conjugate. In this assay, the coloration is enhanced by the binding of two-colored preparations in the test zone, whereas the antigen in the sample inhibits both the binding in the test zone and the enhancing process. The proposed approach, considered in more detail in the Section 3 (see below), is implemented for imidacloprid (IMD) detection in honey as an example.

IMD is a systemic neonicotinoid insecticide widely applied for plant protection [13]. Actually, its use has been of great concern due to its negative effects on nontarget organisms, in particular, honey bees [14]. Collecting nectar and pollen, bees bring IMD into the hives and their own bodies. Chronic exposure to this compound causes developmental abnormalities, weakening of protection against pathogens, deterioration of navigation ability, and so on [14,15]. The use of IMD is associated with a significant decrease in the number of honey bees around the world [16,17,18], which is critical not only for beekeeping as an agricultural sector but also for the pollination of plants and their normal development in natural ecosystems and during cultivation [19,20]. IMD influences the taste, smell, and nutritional value of honey. Moreover, IMD, when it enters the human body, causes allergic reactions, disruption of endocrine and reproductive systems, neurotoxic effects, and so on [21,22,23]. 

With the increasing threat of honey bees’ extinction and the more frequent contamination of honey, sensitive and productive methods of IMD control are in high demand. In modern practice, liquid chromatography with tandem mass spectrometry, gas chromatography with mass spectrometry, high-performance liquid chromatography, and capillary electrophoresis are mainly used for this purpose [24,25]. These methods are highly sensitive and selective, but they require qualified personnel and expensive equipment, and are quite lengthy. Among the immunoassays for IMD, ELISA dominates. This method is relatively simple, but it can be implemented only in laboratory conditions and has a significant duration of several hours [26,27]. Therefore, rapid techniques for IMD immunodetection in honey are needed.

In the article, we consider the preparation and characterization of reagents for the proposed LFIA; the choice of optimal assay conditions; the estimation of its characteristics and comparison with the traditional LFIA, including a consideration of mathematical models; and the approbation of the developed technique for the detection of IMD in honey.

## 2. Materials and Methods

### 2.1. Reagents and Materials

The following compounds were used: goat anti-mouse antibodies, horseradish peroxidase-labeled anti-mouse antibodies (both from Imtek, Moscow, Russia), chloroauric acid, IMD, Tween-20, 3.3′,5.5′–tetramethylbenzidine (TMB), sodium citrate (Sigma-Aldrich, St. Louis, MO, USA), bovine serum albumin (BSA) (Boval Biosolutions, Cleburne, TX, USA), and Triton X-100 (Panreac AppliChem, Chicago, IL, USA). Anti-IMD monoclonal antibodies and IMD–BSA conjugate were from Creative Diagnostics (USA). The manufacturer’s data on the anti-IMD antibodies testing in ELISA demonstrate their high selectivity, manifested as low cross-reactivities with respect to clothianidin (0.5%) and thiacloprid (0.4%) Salts, acids, and alkalis were of analytical or chemical grade. Deionized water cleansed by the Simplicity system (Millipore, Billerica, MA, USA) was applied to prepare gold nanoparticles and their conjugates.

The working nitrocellulose membrane UniSart CN180 (Sartorius, Goettingen, Germany), the PT-R5 conjugate application membrane, the GFB-R7L sample membrane, the AP-045 adsorption membrane, and the L-P25 plastic support (all from Advanced Microdevices, Ambala Cantt, India) formed the set of materials for test strip preparation.

### 2.2. Syntheses of Gold Nanoparticles 

GNPs were synthesized according to the Frens method [28]. First, 100 mL of 0.01% HAuCl_4_ had been brought to boiling. Thereafter, 1.5 mL of 1% sodium citrate was added with vigorous stirring. The mixture was brought to a boil for 15 min, cooled, and then stored at 4 °C.

### 2.3. Conjugation of Anti-IMD Antibodies and IMD–BSA Conjugate with Gold Nanoparticles 

The GNP solution was adjusted to pH 8.5–9.0 with 0.2 M K_2_CO_3_. The anti-IMD antibodies or the IMD–BSA conjugate, dialyzed against a 10 mM Tris–HCl buffer, pH 8.0, were then added to the GNPs at a final concentration of 10 μg/mL. After 45 min of incubation, the solution was mixed in a volume ratio of 40:1 with 10% BSA (so that final concentration of BSA was 2.5 mg/mL). The mixture was stirred for 10 min with Intelli-Mixer RM-2S (ELMI, Riga, Latvia) at 60 rpm. GNPs were precipitated by centrifuging for 15 min at 13,400× *g* and 4 °C. The pellets were collected, twice resuspended in 10 mM Tris, pH 8.5, 1% BSA, and 1% sucrose (TBSA), then centrifuged once more. The finished precipitate was redissolved in TBSA containing 0.05% sodium azide and stored at 4 °C.

### 2.4. Transmission Electron Microscopy

Images of GNPs were obtained using a CX-100 electron microscope (Jeol, Tokyo, Japan) at an acceleration voltage of 80 kV and a magnification of 33,000. The images were scanned and analyzed with the help of the Image Tool program (UTHSCSA, San Antonio, TX, USA).

### 2.5. Conducting Competitive ELISA of Imidacloprid

Into the wells of a Costar microplate (Corning, New York, NY, USA) with immobilized IMD–BSA conjugate (1 μg/mL), IMD at concentrations from 200 to 0.003 ng/mL in 50 mM phosphate buffer, pH 7.4, containing 0.05% Triton X-100 (PBST), was added. Then, anti-IMD antibodies were added at a concentration of 47 ng/mL and incubated for 1.5 h at 37 °C. After the microplate washing with PBST, an anti-species antibodies–peroxidase conjugate was added and incubated for 45 min at 37 °C. The microplate was washed with PBST and the substrate solution—0.4 mM TMB and 1.8 mM H_2_O_2_ in 0.1 M citrate buffer, pH 4.0—was added to the wells. After 15 min incubation at room temperature, the reaction was stopped by addition of 1 M H_2_SO_4_, and the absorbance at 450 nm was measured using a Zenyth 3100 microplate photometer (Anthos Labtec Instruments, Salzburg, Austria).

### 2.6. Preparation of Test Strips

GNP conjugate with anti-IMD antibodies was immobilized on conjugate application membrane from a solution with OD_520_ = 5.0 at 13 μL per cm of the strip. GNP conjugate with IMD–BSA was immobilized on conjugate application membrane from a solution with OD_520_ = 2.5 in the same amount. In a Scheme ‘A’ (see below), an analytical zone was formed with IMD–BSA conjugate; namely, 2 μL of the conjugate (1.0 mg/mL in 50 mM PBS, pH 7.4) was applied per 1 cm of the strip. In Schemes ‘B’ and ‘C’ (see below), 2 μL of the anti-IMD antibodies (1.0 mg/mL in 50 mM PBS, pH 7.4) was applied per 1 cm of the strip. The membranes with the applied reagents were dried at 20–22 °C for 24 h. A multimembrane composite was assembled and cut into 3.5 mm-wide strips using an Index Cutter-1 automatic guillotine cutter (A-Point Technologies, Gibbstown, NJ, USA).

### 2.7. Preparation of Honey Samples

A series of artificially contaminated honey samples were made using at least 15 μL volume of stock IMD solutions for reproducibility. The contaminated honey samples were 5-fold diluted. Namely, 250 mg of honey was mixed with 1 mL of 50 mM phosphate buffer, pH 7.4, containing 0.25% Tween-20 (PBST).

### 2.8. Implementation of LFIA

Assays were performed at room temperature. PBST or honey diluted in PBST (1:4) containing 40–0.2 ng/mL of IMD was added to microplate wells. Test strips were inserted into the wells in a vertical position. Each sample was measured in triplicate and the average staining value was calculated. After 10 min, the strip was removed and placed on a horizontal surface.

### 2.9. Processing Test Strip Images and Calculating Assay Parameters

After LFIA, the test strips were scanned with a Canon Lide 90 flatbed scanner at 600 dpi resolution without contrast and color correction mode, and analyzed with Total Lab software (Nonlinear Dynamics, Newcastle, UK). Line coloring intensities were presented as the same relative units for all data within the article. 

The dependences of coloration intensity in the test zone (for experimental data) or concentration of detected labeled immune complexes (for theoretical data) (y) on the antigen concentration in the sample (x) were approximated by the Origin software version 9.0 (OriginLab, Northampton, MA, USA) using a 4-parameter sigmoid function:

y = (a − b)/[1 + (x/c)^d^] + b,

where a = maximal signal, b = minimal signal, c (or IC_50_) = antigen concentration that inhibits 50% of antibody binding, and d = slope of fitted curve at point c.

The concentration of antigen, corresponding to the disappearance of color in the analytical zone, was taken as the visual detection limit. The instrumental detection limit was calculated from the concentration dependence using 3σ criterion for the difference between the registered coloration and the value for the analyte-free sample [29]. 

## 3. Results

**Figure 1:**
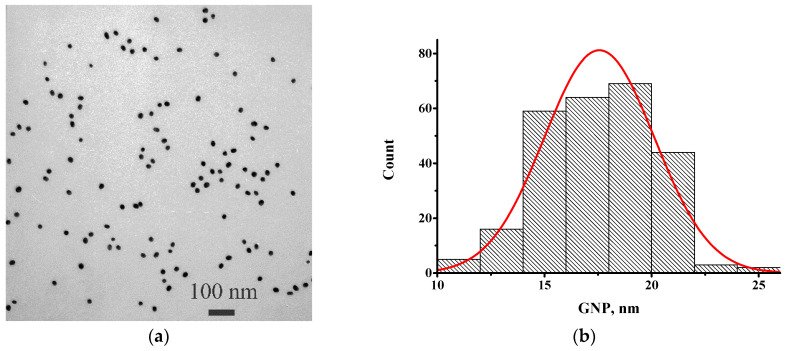
Transmission electron microscopy data for GNPs: (**a**) micrograph; and (**b**) histogram of diameters distribution.

The GNPs were synthesized by the Frens method [28], chosen on the basis of our earlier data [12] and the recommendations of GNPs with diameters in the range of 20–40 nm for the LFIA [30,31]. The resulting GNP preparation was characterized by transmission electron microscopy. According to the obtained data (Figure 1), the average diameter of the nanoparticles was 17.6 ± 2.6 nm (*n* = 262; the minimum value was 10.2 nm and the maximum value was 25.6 nm) with a degree of ellipticity of 1.19 ± 0.17.

### 3.1. Characterization of Immunoreagents

**Figure 2:**
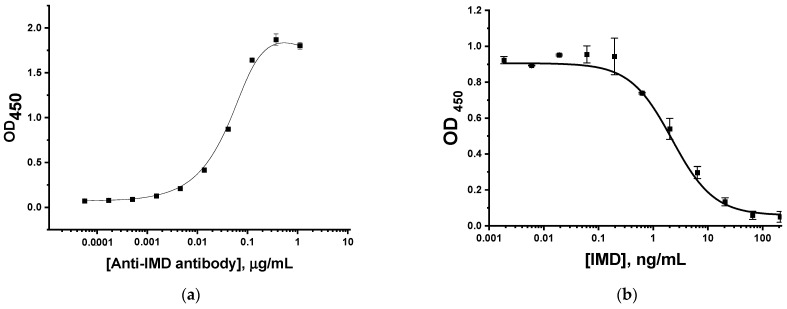
Testing immunoreactants for IMD in ELISA: (**a**) binding anti-IMD antibody with immobilized IMD–BSA conjugate; and (**b**) competitive detection of IMD.

An enzyme immunoassay was used to assess the reactivity of monoclonal antibodies to IMD. The study of their binding with the immobilized IMD–BSA conjugate is presented in Figure 2a. The obtained dependence allowed us to choose the antibody concentration for the competitive assay as the value providing an OD_450_ = 1.0 and, in this way, a good amplitude of competitive curve. Thus, we chose the antibody concentration equal to 47 ng/mL and realized the competitive ELISA with its use. As can be seen in Figure 2b, the limit of detection in the competitive ELISA of IMD was 1 ng/mL, and the operating range was 0.7–9 ng/mL. Thus, the immunoreagents are suitable for use in LFIA development.

### 3.2. Mathematical Study of Competitive LFIA Schemes

The common competitive LFIA (Scheme ‘A’) uses three reagents: antibodies conjugated with marker particles (P), analyte-competitor (A), and receptor molecules immobilized in the test zone—as a rule, carrier protein–antigen conjugate (R). The increasing of the analyte concentration A reduces the free concentration P, which suppresses the formation of PR in the second reaction. The concentration of the PR complex reflects the amount of the label bound in the test zone and, accordingly, determines the signal intensity. The vast majority of competitive LFIAs are based on this principle. 

**Figure 3:**
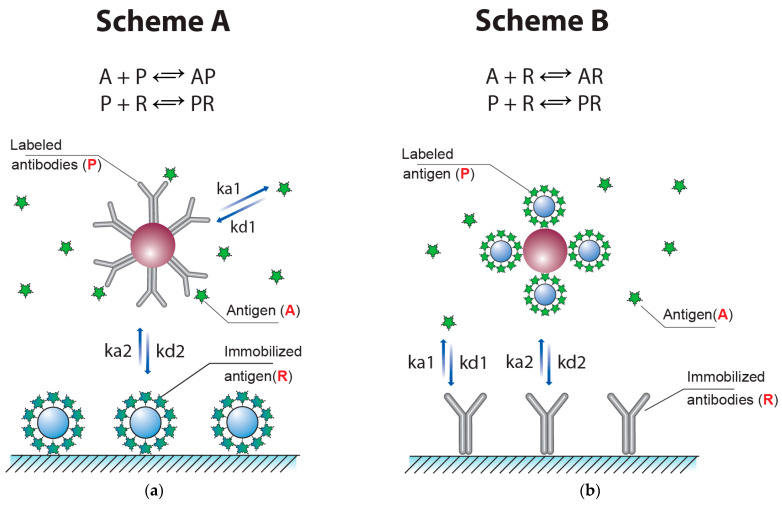
Competitive LFIAs with one process of competition. Scheme ‘A’ with labeled antibodies (**a**) and Scheme ‘B’ with labeled antigen (**b**).

However, there is a variation of the described scheme, in which an antigen is labeled and antibodies are immobilized in the test zone [32,33]. Examples of developed test systems for the LFIA that realized Scheme ‘B’ can be found in [34,35,36]. This scheme (Scheme ‘B’) is also described by two reactions, A + R = AR and P + R = PR, where P is the labeled antigen and R is the immobilized antibodies (Figure 3b).

**Figure 4:**
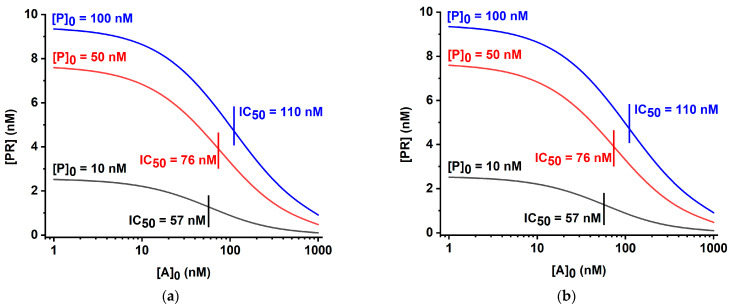
Theoretical calibration curves for Schemes ‘A’ (**a**) and ‘B’ (**b**) of competitive LFIA at various initial concentrations of the labeled reagent [P]_0_. Initial concentrations [R]_0_ are 10 nM, kinetic association constants of all immune interactions are 10^5^ M^−1^ s^−1^, and kinetic dissociation constants of all immune interactions are 10^−4^ s^−1^.

The analytical parameters of these systems under varied concentrations and affinities of the immunoreactants were considered theoretically for algebraic solutions [32,33] and have been tested in our study using a numerical calculation in the created COPASI-based digital models (https://copasi.org/ (accessed on 23 March 2023)) for a better comparison with the developed, more complicated LFIA based on double competition and double labeling. As can be seen from Figure 4, in both cases, variation of the ratio of the labeled and immobilized reagents led to integral changes of the maximal coloration intensity and detection limit. A decrease in the concentration of the labeled reagent caused both a lowering of the detection limit and less intense coloration of the test zone in the absence of an analyte. These theoretical data are in accordance with the known experimental characterizations of the competitive LFIA [37,38]. Thus, obtaining a more sensitive test by reducing [P]_0_—the initial concentration of P (subscript index 0 hereinafter denotes the initial concentration)—is accompanied by weak coloration and, consequently, a complicated interpretation of the assay results and less accurate calculations of the base of the calibration curve.

To reach the lower detection limits of the competitive LFIA without a significant loss of coloration intensity, we earlier proposed using two competitive interactions instead of one [12]. For this, labeled antibodies (P) are used, and the same antibodies without a label (R) are immobilized in the test zone. The sample is mixed with a conjugate containing a protein carrier and several antigen molecules coupled to it (C). In this system, the generation of the detected colored complex PCR is inhibited by the analyte in the sample (A) via its interaction with the labeled and immobilized antibodies, that is, via two competitions with C. Unlike common LFIAs (both Schemes ‘A’ and ‘B’), in the described scheme, the presence of the analyte in the sample leads to a sharper decrease in color, and the detection limit decreases.

**Figure 5:**
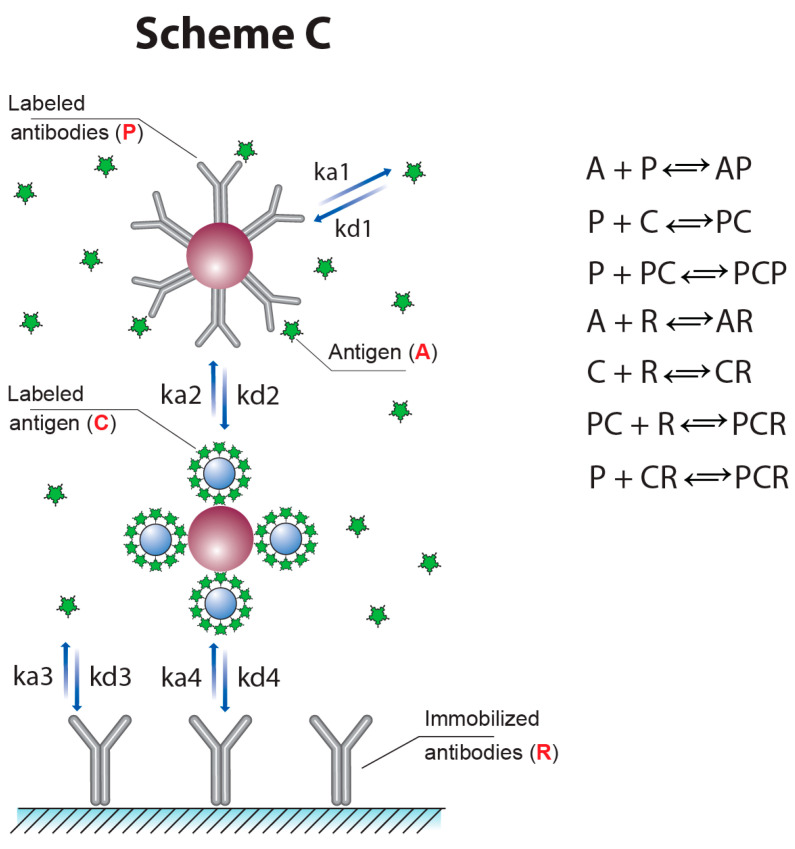
Scheme ‘C’ of competitive LFIA with two labeled reagents.

However, the assay scheme described in [12] does not provide the possibility for a simultaneous increase in the signal value and decrease in the detection limit. In this work, a new approach was proposed and characterized with the inclusion of the detected label into the reagent C, and this was realized by both double competition and double labeling. The interactions in the novel scheme (Scheme ‘C’) are presented in Figure 5. Note that the detected signal in this case accords with the sum of the concentrations of the CR complex and the doubled concentrations of the PCR complex. Thus, the use of two GNP conjugates allows us to extend the variants to incorporate labels into the complexes formed in the test zone.

**Figure 6:**
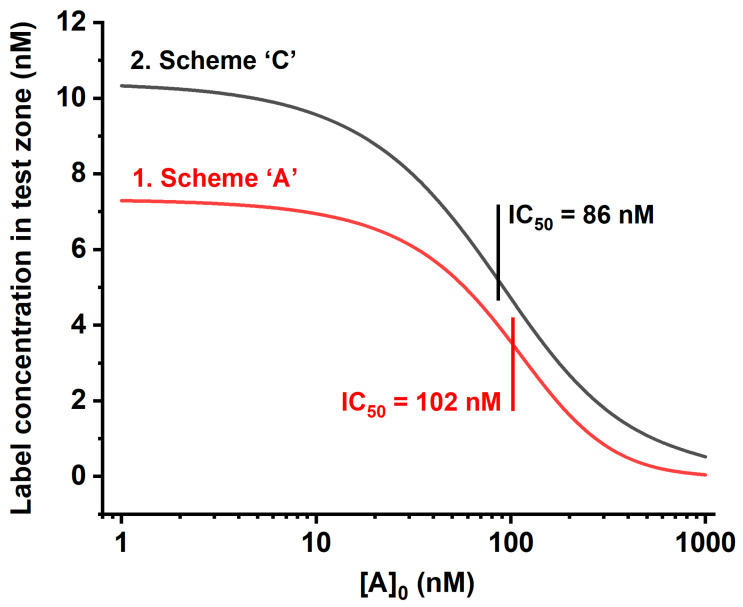
Theoretical calibration curves of competitive LFIA in Schemes ‘A’ and ‘C’: (1) value [PR] for Scheme ‘A’; and (2) value 2 × [PCR] + [CR] for Scheme ‘C.’ Initial concentrations for Scheme ‘A’: [R]_0_ = 10 nM, and [P]_0_ = 50 nM; for Scheme ‘C’: [R]_0_ = 10 nM, and [P]_0_ = [C]_0_ = 50 nM. Kinetic association constants of all immune interactions are 10^5^ M^−1^ s^−1^; kinetic dissociation constants of all immune interactions are 10^−4^ s^−1^.

Our numerical modeling for Scheme ‘C’ demonstrated that it provides a higher signal with a lower detection limit than the most widespread scheme, Scheme ‘A.’ Figure 6 gives the theoretical calibration dependences of the [PR] and 2 × [PCR] + [CR] values, reflecting the signal in the test zones for Schemes ‘A’ and ‘C,’ respectively. It can be seen that the binding of two kinds of labeled reactants in the test zone leads both to an increase in coloration and to a shift in the inflection point of the calibration dependence to lower concentrations, which is accomplished by reaching the lower detection limit. This is due to the fact that in the proposed LFIA format, the presence of a competitor (analyte) suppresses both reactions: the binding of antibodies to the labeled antigen and the binding of the resulting complex to the labeled antibodies.

### 3.3. Choice of Conditions for the LFIA with Two Conjugates (Scheme ‘C’)

Because the immunoreagents are in complexes with GNPs, the optimal conditions for implementing the proposed scheme were determined by varying the concentrations of GNP-specific antibodies and GNP–hapten–protein conjugates. To do this, we studied the effect of conjugates with different optical densities at 520 nm on the signal generation in the test zone.

**Figure 7:**
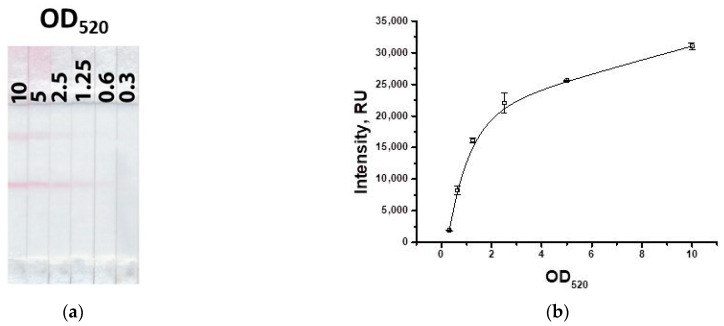
Appearance of test strips for varying concentration of the GNP–hapten–protein conjugate (**a**) and the dependence of coloration intensity in the test zone on the concentration of the GNP–IMD–BSA conjugate (**b**).

As can be seen from Figure 7, as the concentration of the GNP–IMD–BSA conjugate decreases, the signal decreases. The concentration of the conjugate with OD_520_ = 2.5 was chosen, at which the signal differs slightly from higher concentrations and provides a coloration of the test zone acceptable for visual detection.

**Figure 8:**
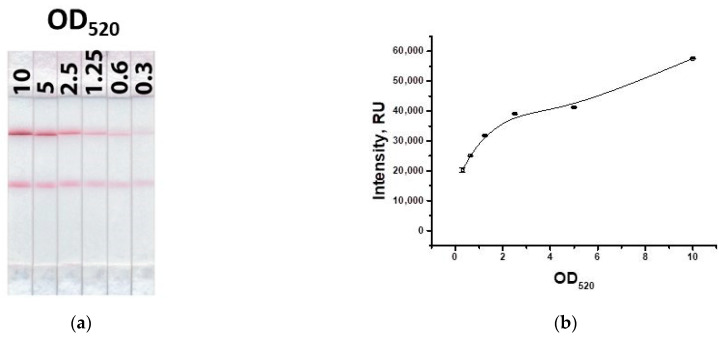
Appearance of test strips for varying concentration of the GNP–antibody conjugate (**a**) and the dependence of coloration intensity in the test zone on the concentration of the GNP–antibody conjugate (**b**).

Next, the concentration of the GNP-specific antibody conjugate was determined upon binding in the test zone, taking into account the fixed concentration of the GNP–IMD–BSA conjugate. Figure 8 shows that the use of the second conjugate leads to an increase in the signal by an average of two times. Because the concentration of the conjugate with specific antibodies should be higher than that of the conjugate with the hapten–protein to ensure low detection limits, an OD_520_ = 5 was chosen.

### 3.4. Characterization of IMD LFIA

**Figure 9:**
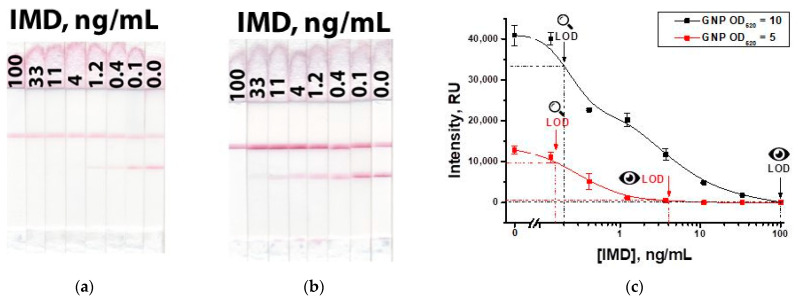
LFIA of IMD in the common Scheme ‘A’ with OD_520_ = 5 (**a**) of GNP-anti-IMD antibody and OD_520_ = 10 (**b**) of GNP-anti-IMD antibody; and calibration curves for both variants (**c**).

According to the chosen parameters, IMD was determined in PBST. Figure 9 shows the results of a common Scheme ‘A’ of the LFIA. The visual detection limit was 4 ng/mL at a conjugate OD_520_ = 5 and 100 ng/mL at a conjugate OD_520_ = 10. 

The main disadvantages of the standard competitive LFIA are high detection limits with a low signal in the test zone, which makes it difficult to interpret the assay results. To provide a higher signal, it is necessary to increase the concentration of the label conjugate. However, an interconnected increase in the concentration of antibodies leads to a higher detection limit.

**Figure 10:**
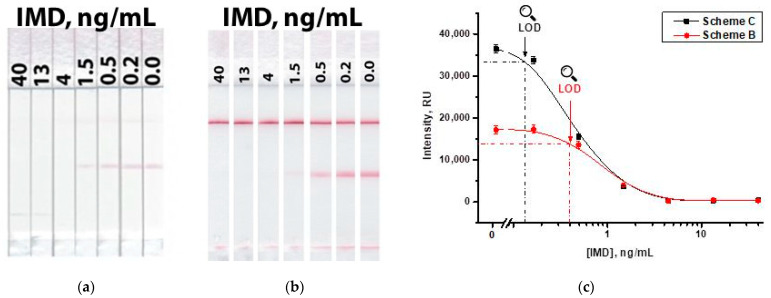
LFIA of IMD in Scheme ‘B’ (**a**) and in Scheme ‘C’ (**b**); and calibration curves for both schemes (**c**).

The use of a two-conjugate Scheme ‘C’ overcomes these shortcomings, demonstrating the retention of a high signal and a low detection limit. Figure 10 shows the results of the IMD detection according to Scheme ‘B’ and in the proposed Scheme ‘C.’ As can be seen, while maintaining the same values of the detection limit (4 ng/mL), the signal in the test zone for the scheme with two conjugates approximately doubles (Figure 10c), which contributes to a more reliable interpretation of the results. The instrumental detection limits in Schemes ‘B’ and ‘C’ were 0.4 and 0.13 ng/mL, respectively. The concentration dependences of coloration had linear ranges of 0.5–1.5 ng/mL for Scheme ‘B’ and 0.2–1.5 ng/mL for Scheme ‘C’.

### 3.5. Approbation of LFIA for Honey Sample Testing

Due to the significant viscosity of honey, its samples cannot be tested in the LFIA as is. At the same time, the greater the dilution of the samples before the LFIA, the higher the minimum antigen content detected in a gram of the initial honey sample. Therefore, the testing of honey samples was preceded by the choice of their optimal dilution. Samples diluted two, five, and 10 times with PBST were characterized. A reproducible and uniform movement of the liquid front along membranes of the test strip was observed starting from a five-fold dilution, which was chosen for further work.

**Figure 11:**
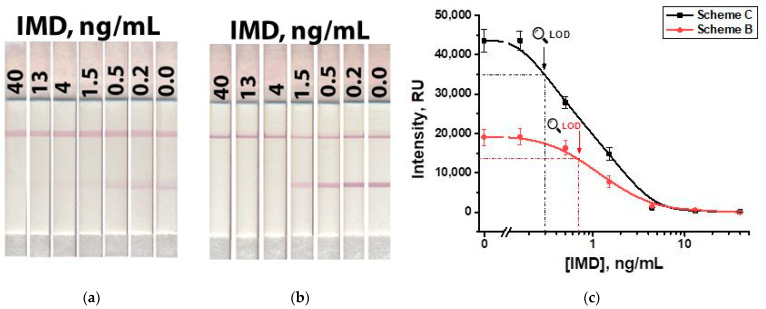
LFIA of IMD in honey in Scheme ‘B’ (**a**) and Scheme ‘C’ (**b**); and calibration curves for both schemes (**c**).

We have tested artificially contaminated honey samples containing IMD at concentrations from 40 to 0.2 ng/mL, five-fold-diluted with PBST. The visual limit of detection, corresponding to the disappearance of coloration in the test zone, was 4 ng/mL (16 µg/kg in conversion per weight of honey) for Schemes ‘B’ and ‘C,’ respectively (Figure 11). With instrumental registration, the limits of detection of IMD in honey were 0.7 and 0.3 ng/mL (2.8 µg/kg and 1.2 µg/kg in conversion per weight of honey) for Schemes ‘B’ and ‘C,’ respectively. It exceeds the MRL of IMD in honey (50 µg/kg) [39]. Calibration curves (Figure 11c) demonstrated the maintaining of the same linear ranges as with the measurements in the buffer (see Figure 10c), namely, 0.5–1.5 ng/mL for Scheme ‘B’ and 0.2–1.5 ng/mL for Scheme ‘C’. These ranges accord with an IMD content in honey of 2.0–6.0 µg/kg for Scheme ‘B’ and 0.8–6.0 µg/kg for Scheme ‘C’.

The testing of spiked honey samples (three tested concentrations with three repetitions) demonstrated that the revealing of IMD in honey was in the range of 87–92% (Table 1).

Considering the existing developments of the LFIA for IMD, we can conclude that the assays that are based on a common competitive scheme and GNPs as labels are characterized by detection limits in the range of 10–50 ng/mL [40,41,42,43]. Reaching improved sensitivity to IMD in the LFIA (0.01–0.79 ng/mL) [44,45,46,47] is associated with label changes, a more complicated assay realization with additional reactants, or using special instrumentation such as the time-resolved fluorimeter.

The presented development makes it possible to achieve low sensitivity without alternate labels and additional reagent stages. It demonstrates that the simultaneous use of two conjugates in the competitive LFIA—a labeled analyte derivative and labeled antibodies—allows us to overcome the limitations of the standard competitive scheme.

## 4. Conclusions

To detect the insecticide imidacloprid in honey samples, the traditional scheme of the competitive lateral flow immunoassay and the developed scheme with double competition and two markers were used. The conducted experimental comparison gives grounds for the following conclusions:

The instrumental detection limit of the developed scheme was 1.2 µg/kg, which is 2.3 times less than in the traditional scheme. The proposed scheme is characterized by a high sensitivity and higher signal compared to the traditional scheme.

To implement the scheme with double competition and two markers, a larger number of reagents and a more complex procedure for making test strips are required. However, when using the prepared test strips, no complications are observed. By pre-applying all the necessary reagents to the corresponding zones of the test strip, the analysis procedure is simplified to immersing the cut of the test strip into the sample. After that, all the processes necessary for the formation of the detected complexes are initiated by the spontaneous movement of the liquid along the test strip and do not require any additional actions from the operator.

Note that despite the greater number of reagents involved in analytical interactions, the reproducibility of repeated measurements for the proposed scheme did not increase reliably.

The time of the assay is determined by the speed of reagents’ movement along the test strip. In this regard, the time does not differ for the two assay formats, being 10 min in both cases.

An important feature of the proposed scheme with double competition and two markers is its versatility, that is, the possibility of implementing various low-molecular-weight compounds for the competitive LFIA. Therefore, potential future prospects in the study of this approach and its applications can be the development of test systems for other practically significant compounds, the characterization of changes in the detection limit, and the working range of the LFIA when using it.

Another interesting task for further studies is the evaluation of the proposed new LFIA format in cases of class-selective detection as a tool to modulate the cross-reactivity values of structurally similar compounds in comparison with the common LFIA format. The possibility of such modulations by a simple variation of reactant concentrations was demonstrated in our earlier study [48] and can be integrated with the double competition technique.

## Figures and Tables

**Table 1:** Recoveries of IMD in honey samples by LFIA in Scheme ‘C’.

IMD Added, ng/mL	IMD Detected ± SD, ng/mL	Recovery, %
0.2	0.18 ± 0.02	90
0.5	0.46 ± 0.05	92
1.5	1.30 ± 0.05	87

## Data Availability

The data that support the findings of this study are available from the corresponding author upon request.
